# Plants changed the response of bacterial community to the nitrogen and phosphorus addition ratio

**DOI:** 10.3389/fpls.2023.1168111

**Published:** 2023-03-27

**Authors:** Zehao Zhang, Jingkuan Sun, Tian Li, Pengshuai Shao, Jinzhao Ma, Kaikai Dong

**Affiliations:** ^1^ Shandong Key Laboratory of Eco-Environmental Science for Yellow River Delta, Binzhou University, Binzhou, China; ^2^ College of Forestry, Shandong Agricultural University, Taian, China

**Keywords:** *Phragmites communis*, bacterial community, Yellow River Delta, nitrogen to phosphorus ratio, rhizosphere soil

## Abstract

**Introduction:**

Human activities have increased the nitrogen (N) and phosphorus (P) supply ratio of the natural ecosystem, which affects the growth of plants and the circulation of soil nutrients. However, the effect of the N and P supply ratio and the effect of plant on the soil microbial community are still unclear.

**Methods:**

In this study, 16s rRNA sequencing was used to characterize the response of bacterial communities in *Phragmites communis* (*P.communis*) rhizosphere and non-rhizosphere soil to N and P addition ratio.

**Results:**

The results showed that the a-diversity of the *P.communis* rhizosphere soil bacterial community increased with increasing N and P addition ratio, which was caused by the increased salt and microbially available C content by the N and P ratio. N and P addition ratio decreased the pH of non-rhizosphere soil, which consequently decreased the a-diversity of the bacterial community. With increasing N and P addition ratio, the relative abundance of *Proteobacteria* and Bacteroidetes increased, while that of *Actinobacteria* and *Acidobacteria* decreased, which reflected the trophic strategy of the bacterial community. The bacterial community composition of the non-rhizosphere soil was significantly affected by salt, pH and total carbon (TC) content. Salt limited the relative abundance of *Actinobacteria*, and increased the relative abundance of *Bacteroidetes*. The symbiotic network of the rhizosphere soil bacterial community had lower robustness. This is attributed to the greater selective effect of plants on the bacterial community influenced by nutrient addition.

**Discussion:**

Plants played a regulatory role in the process of N and P addition affecting the bacterial community, and nutrient uptake by the root system reduced the negative impact of N and P addition on the bacterial community. The variations in the rhizosphere soil bacterial community were mainly caused by the response of the plant to the N and P addition ratio.

## 1 Introduction

Atmospheric N deposition has continuously increased caused by human activities and fossil fuel burning. The soil N content in Chinese natural ecosystems increased by 8 Kg N ha^-1^yr^-1^ from 1980 to 2000 (Liu et al., 2013). According to the current trend, N deposition will increase to double the current level (Peñuelas et al., 2012; Lu et al., 2021). P is mainly from the rock, which is different from N mainly stored in the atmosphere (Dong et al., 2019). In addition, ecosystems obtain P mainly through the decomposition and mineralization of soil organic matter (SOM) by soil extracellular enzymes (Bell et al., 2014; Dong et al., 2019). The lagged and low input of P compared to multiple pathways of N input (e.g., atmospheric N deposition, microbial mineralization) has resulted in a gradual imbalance of the N and P input ratio (Deng et al., 2017; Dong et al., 2019). N and P input must be coordinated and synergistic because organisms need a relatively constant ratio of elements to metabolize (Elser et al., 2007). Imbalance of N and P input may cause soil degradation (Zhu et al., 2016), which may affect the regular function of the bacterial community and thus cause structural changes and dysfunction of the ecosystem (Cusack et al., 2011; Tian and Niu, 2015).

Microorganisms are the most diverse and abundant organisms on the planet and are essential to the nutrient cycle (Bennett et al., 2017). Extensive reports have simulated the effects of N deposition on soil microbial communities through N addition experiments, but the conclusions are controversial. On the one hand, the increase in soil N content due to N addition promotes the growth of the bacterial community and increases the diversity of the bacterial community (Fierer et al., 2012; Redding et al., 2016). On the other hand, soil acidification and low pH caused by N addition makes the bacterial community in an unfavorable environment for growth (Sha et al., 2021). Compared to N addition, there are relatively few P addition studies. P addition had a significant effect on the α-diversity and composition of the soil bacterial community (Ma et al., 2021). However, it has also been found that the effect of P input on the bacterial community is negligible (Widdig et al., 2020; Ran et al., 2021). These works have focused on the effect of single nutrient input on the soil bacterial community. Wang et al. (2018b) found that N and P input played different roles in influencing soil bacterial richness and community. The negative effects of N and P imbalance have been observed in the study of plant community structure and diversity (Liu et al., 2019). Li et al. (2022a) found that N and P imbalance input changed microbial biomass. However, the effect of the N and P addition ratio on α-diversity and structure of the bacterial community is poorly understood. The N addition can lead to the P limitation (Zhu et al., 2016; Mao et al., 2017). As imbalances continue to tilt in the same direction, the effects of imbalances will continue to increase (Penuelas and Sardans, 2022). Therefore, the effect of the N and P addition ratio on the bacterial community is more important to study than single nutrient.

Previous studies have found that the effect of N addition on the bacterial community is regulated by the plant. Wang et al. (2022a) found that the plant rhizosphere soil bacterial community differed from the non-rhizosphere bacterial community in response to N addition. Nutrient addition stimulates the release of more C content from plant roots (Liu et al., 2016), which facilitates the growth of bacterial community (Fierer et al., 2009). However, it was also found that although nutrient addition increased the C content released by the root system (Peng et al., 2017), it also increased the proportion of recalcitrant C, which was detrimental to the acquisition and utilization of the bacterial community (Freedman et al., 2013; Liu et al., 2016). Therefore, the study of the effect of nutrient input on the bacterial community should consider the role of plants. It is reasonable to study the plant rhizosphere and non-rhizosphere soil bacterial community separately (Miki et al., 2010).

The Yellow River Delta, located on the southern coast of Bohai Bay, is the youngest and most complete coastal wetland ecosystem in the warm zone, which has important value in maintaining biodiversity, purifying pollutants and regulating microclimate (Liu et al., 2021). During the growing season (MayN-November) in 2012, the nitrogen deposition in the Yellow River Delta was relatively high in the country, at 22.64 Kg ha^-1^ (Yu et al., 2014). Due to the fragility of coastal wetland ecosystems, imbalances of N and P input may be more damaging to the ecosystem. To investigate the effects of N and P addition on bacterial community, control experiments with different N and P addition ratios were designed to research (a) the effect of N and P input ratios on bacterial community, (b) the effect of plants in the process of bacterial community response to N and P ratios, and (c) the coupling correlation between soil and bacterial community by 16s sequencing technology.

## 2 Materials and methods

### 2.1 Study area

This experimental study area is located in the Yellow River Delta wetlands (E 118°44’2”, N 38°1’18”). The annual precipitation is 550-640mm, and the average annual temperature is 12.6°C. The plants are mainly dominated by halophytes, including *Limonium bicolor*, *P.communis*, *Suaeda salsa*, *Tamarix chinensis Lour*, etc. Based on the distribution of vegetation and growth conditions in the study area, etc., we selected the *P.communis* distribution area with the widest distribution range for the experiment. The total area of the experimental area is approximately 120 m^2^.

### 2.2 Experimental design

The N-P ratio was referred to previous studies (Liu et al., 2019; Li et al., 2022a). Five N and P addition treatments were set up, N0P0 (0 g/m^2^ N, 0 g/m^2^ P), N0P1 (0 g/m^2^ N, 1 g/m^2^ P), N1P1 (5 g/m^2^ N, 1 g/m^2^ P), N2P1 (15 g/m^2^ N, 1 g/m^2^ P), N3P1 (45 g/m^2^ N, 1 g/m^2^ P). Use urea and KH_2_PO_4_ as N and P fertilizers respectively (; Li et al., 2019a). Urea and KH_2_PO_4_ were dissolved in pure water and then divided equally into 4 parts. From May to August 2021, N and P addition were added at the beginning of each month. Three replicates were set up for each treatment. A 1 m buffer interval was set up to prevent nutrient flow.

### 2.3 Soil sampling

Soil collection was conducted in September 2021. Dig out the *P.communis* completely, shake off the loose soil and then collect the soil attached to the root with the brush, which is considered as rhizosphere soil. In treatment samples, the soil of 5-10 cm depth with no plant growth was collected as non-rhizosphere soil. The collected soil was divided into two parts. One part was used for measuring soil physical and chemical properties, and the other part was used for microbial sequencing.

### 2.4 Measurements of soil physical and chemical properties

Total nitrogen (TN) and total carbon (TC) content were measured by elemental analyzer (Vario EL III, Elementar, Germany). Total phosphorus (TP) and available phosphorus (AP) content were measured by the ultraviolet-visible spectrophotometer. Soil pH was measured by the pH meter. SOM content was measured by the potassium dichromate volumetric method. The salt content was measured by the conductivity method.

### 2.5 Soil DNA extraction, sequencing and data analysis

Genomic DNA was extracted from the samples using the soil DNA kit (Omega, USA), followed by agarose gel electrophoresis to detect the purity and concentration of DNA, and the appropriate amount of sample was taken in a centrifuge tube and diluted with sterile water. 16S rRNA genes of distinct regions (V3-V4) were amplified used 341F (5’-CCTAYGGGRBGCASCAG-3’) and 806R (5’-GGACTACNNGGGTATCTAAT-3’) primers. PCR products were detected by electrophoresis using agarose gels with 2% concentration and then were purified with Qiagen Gel Extraction Kit (Qiagen, Germany). The MiSeqPE300 platform of Illumina company (Wekemo Tech Group Co., Ltd. Shenzhen China) was used for high-throughput sequencing.

### 2.6 Statistical analysis

The effects of N and P addition ratio on α-diversity, soil environmental factor and dominant bacterial phyla were evaluated by one-way ANOVA analysis followed by LSD multiple range tests. Principal coordinate analysis (PCoA) based on the Bray-Curtis distance of OTUs was performed the difference of bacterial community structure. PERMANOVA was conducted to test the effect of N, P and soil position on bacterial community structure. Aggregated boosted tree (ABT) was used to assess the importance of environmental factors on the α-diversity of the bacterial community. OTUs with relative abundance less than 0.1% are filtered out during network construction. The threshold of edge in the network is absolute value of correlation coefficient (Corr) > 0.6 and P < 0.01. The network hubs were selected based on Within-module connectivity (Zi) > 2.5 or Among-module connectivity (Pi) > 0.62 (Wang et al., 2022a). We observe the rate of network stability degradation by removing a certain percentage of OTUs. The structural equation model (SEM) has been revised several times and has the perfect fit. Redundancy analysis (RDA) was used to analyze the effect of soil on the bacterial community.

## 3 Results

### 3.1 α-diversity analysis

**Figure 1:**
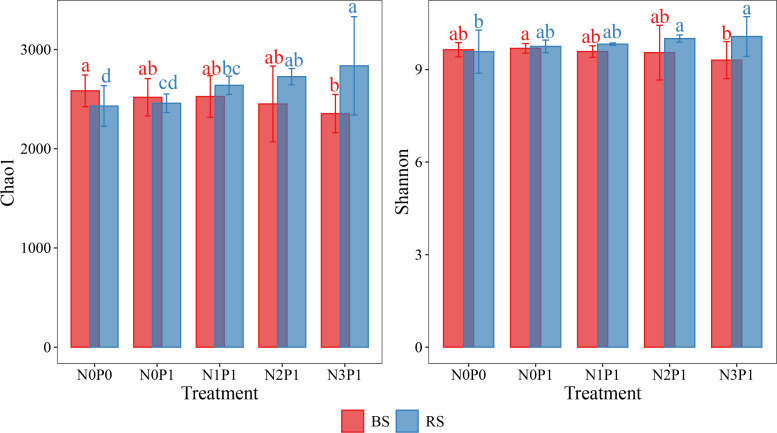
The α-diversity in different N and P addition ratio treatments. BS, non-rhizosphere soil. RS, rhizosphere soil. Different letters with the same color represent significant differences between treatments.

The response of α-diversity of the bacterial community in *P.communis* rhizosphere soil and non-rhizosphere soil differed with changes in N and P addition ratio (**Figure 1**). The α-diversity of the rhizosphere soil bacterial community increased with N and P ratio increasing and reached a maximum at the N3P1 treatment. The Chao1 index significantly increased by 16.7% (P < 0.05) and 15.4% (P < 0.05) compared to N0P0 and N0P1 treatments, respectively, and the Shannon index significantly increased by 5.1% compared to N0P0 treatment. In the non-rhizosphere soil, bacterial community α-diversity decreased with increasing N and P ratio. Compared to the N0P0 treatment, the Chao1 index decreased by 8.7% in the N3P1 treatment (P < 0.05). The α-diversity of the two soil bacterial communities was significantly different in the N3P1 treatment (**Table S1**, P < 0.05). The analysis combining soil position, N and P addition revealed that the α-diversity of the bacterial community was significantly affected by soil position (**Table S2**, P < 0.01). The interaction between N addition and soil position also had a significant effect on bacterial community α-diversity (**Table S2**, P < 0.01), indicating that the effect of N addition on bacterial community α-diversity was related to soil position.

### 3.2 PCoA analysis

**Figure 2:**
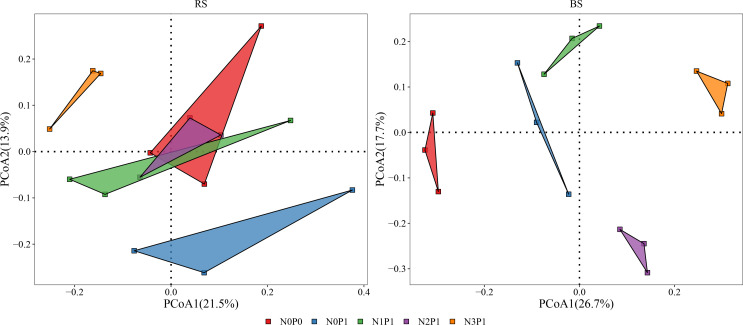
The PCoA analysis in different N and P addition ratio treatments. BS, non-rhizosphere soil. RS, rhizosphere soil.

PCoA analysis of bacterial community structure is shown in **Figure 2**. The first and second axes explained 21.5% and 13.9% of the variations in the bacterial community of rhizosphere soil, respectively. In the rhizosphere soil, N0P0, N1P1 and N2P1 treatments clustered together, indicating that the bacterial community structure of these three treatments was similar and differed from N3P1 and N0P1 treatments. In non-rhizosphere soil, the first two axes explained 26.7% and 17.7% of the variation in the bacterial community, respectively. The bacterial community was clustered according to treatment, indicating that the N and P addition ratio had a significant effect on the bacterial community structure of non-rhizosphere soil. N and P addition ratio increased the differences in bacterial community structure. N and P had a significant effect on the bacterial community structure of non-rhizosphere soil (**Table S3**, P < 0.05). Nitrogen, phosphorus, and soil position as well as the interaction of all three had significant effects on bacterial community structure (**Table S4**, P < 0.05). Soil position and the interaction of N and soil position had the greatest effect on bacterial community structure, explaining 15.1% and 15.3% of the variation in bacterial community, respectively (P < 0.001).

### 3.3 Bacterial community composition

**Figure 3:**
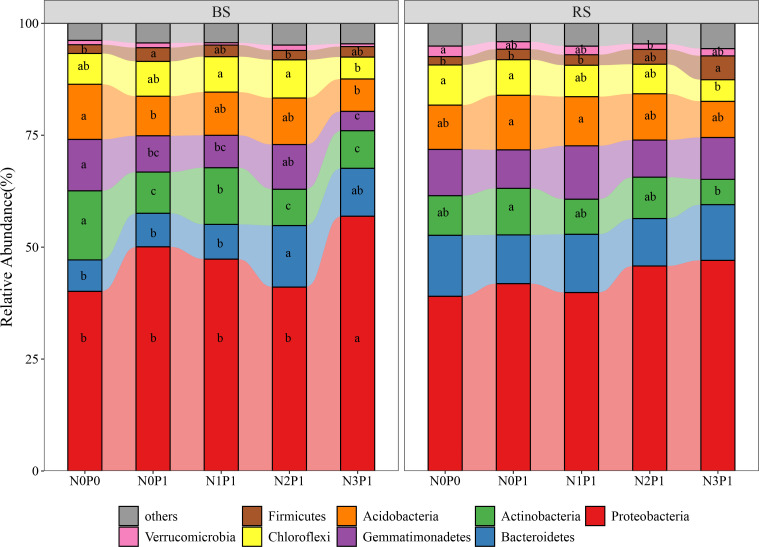
The relative abundance of dominant bacterial phyla in different N and P addition ratio treatments. BS, non-rhizosphere soil. RS, rhizosphere soil. Different lowercase letters represent significant differences between treatments in the same soil position.

The bacterial phylum with relative abundance more than 1% in the rhizosphere and non-rhizosphere soil of *P.communis* is shown in **Figure 3**. In the non-rhizosphere soil, N3P1 treatment significantly increased the bacterial community *Proteobacteria* by 39.9% and 20.6% relative to N0P0 and N0P1 treatment, respectively (P < 0.05). The relative abundance of *Actinobacteria*, *Acidobacteria* and *Gemmatimonadetes* was highest in N0P0 treatment and lowest in N2P1, N3P1 and N3P1 with a decrease of 50.3, 42.3% and 62.6%, respectively (P < 0.05). The *Firmicutes* had the highest relative abundance in the N0P1 treatment, which was significantly higher than the N0P0 treatment (P < 0.05). There was insignificant variation in the *Proteobacteria* and *Bacteroidetes* in the rhizosphere soil. The relative abundance of *Acidobacteria* and *Actinobacteria* was highest in the N0P1 treatment. The relative abundance of both of *Acidobacteria* and *Actinobacteria* decreased with increasing N and P addition ratio, decreasing by 33.6% and 45.2% in the N3P1 treatment, respectively (P < 0.05). The relative abundance of *Chloroflexi* and *Firmicutes* showed contrasting results, with the highest and lowest in N0P0 treatment, respectively. The relative abundance of both decreased and increased with increasing N and P addition ratio, reaching the minimum and maximum value at N3P1 treatment, decreasing and increasing by 46.7% and 178.9%, respectively (P < 0.05).

### 3.4 Symbiotic network analysis

**Figure 4:**
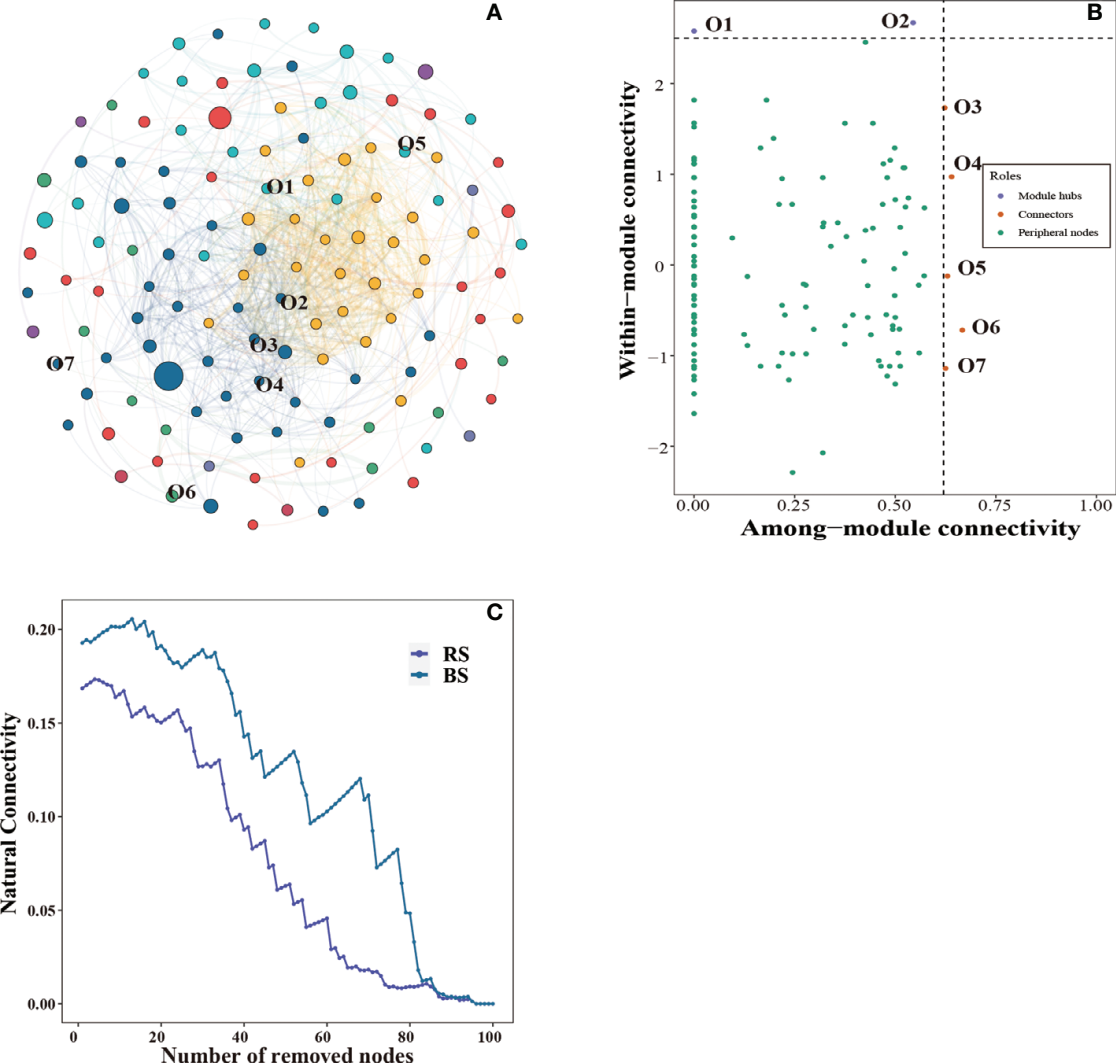
Co-occurrence networks of the bacterial community **(A)**. Putative keystone OTUs **(B)**. The robustness of microbial network for rhizosphere soil and non-rhizosphere soil **(C)**. BS, non-rhizosphere soil. RS, rhizosphere soil.

The bacterial community symbiotic network is shown in **Figure 4A**, and the bacterial community network was divided into 7 modules. Seven key nodes were screened, including five connectors and two module bubs (**Figure 4B**). In addition, we constructed sub-networks for rhizosphere and non-rhizosphere soil, and the number of nodes, number of edges, and complexity of non-rhizosphere soil were higher than those of rhizosphere soil (**Table S5**). To further compare the stability of the networks, we calculated the network robustness based on the natural connectivity. The results showed that the natural connectivity decreased with the removal of more OTU nodes. The network connectivity of rhizosphere soil was significantly lower than that of non-rhizosphere soil (**Figure 4C**). Annotated analysis of key OTUs in the microbial interactions network (**Figure S1**) revealed that seven key OTUs belonged to four phyla, which identified the *Proteobacteria*, *Bacteroidetes*, *Gemmatimonadetes*, and *Actinobacteria*. *Longimicrobiales* order, *Pseudomonas* order, *Cyclobacteriaceae* family and *MND1* genera were significantly higher in rhizosphere soil than in non-rhizosphere soil (**Figure S1**, P < 0.05).

### 3.5 Soil environmental factor analysis

Salt, pH, SOM and TC content were significantly higher in rhizosphere soil than in non-rhizosphere soil (**Table 1**, P < 0.05), while TN and TP showed the opposite (P < 0.05). The soil SOM content increased with increasing N and P addition ratio and was lowest in the N0P1 treatment. In the rhizosphere soil, soil salt content increased with increasing N and P addition ratio, reaching a maximum in the N3P1 treatment, increasing by 57.2% and 40.8% compared to N0P0 and N0P1 treatment, respectively (P < 0.05). The N3P1 treatment decreased pH by 0.19 units compared to the N0P0 treatment (P < 0.05). The pH decreased significantly with increasing N and P ratio in the non-rhizosphere soil (P < 0.05).

**Table 1:** The soil environmental factors of bacterial community in different N and P addition ratio treatments.

Soil position		N0P0	N0P1	N1P1	N2P1	N3P1
BS	TC^B^	22.82 + 0.43b	24.58 + 0.76ab	25.34 + 1.75a	26.39 + 1.11a	25.9 + 0.77a
	SOM^B^	16.53 + 0.62a	9.22 + 0.82c	12.36 + 1.2b	12.16 + 1.21b	15.68 + 0.98a
	AP	7.31 + 3.29c	14.15 + 3.95b	20.84 + 3.23a	11.37 + 2.45bc	11.68 + 1.06bc
	pH^B^	7.79 + 0.05a	7.78 + 0.14a	7.65 + 0.04b	7.6 + 0.02b	7.57 + 0.01b
	Salt^B^	1.47 + 0.11d	1.81 + 0.14c	2.39 + 0.1b	2.6 + 0.09a	2.55 + 0.04ab
	TP^A^	0.71 + 0.01ab	0.74 + 0.05ab	0.78 + 0.02a	0.7 + 0.08b	0.72 + 0.02ab
	TN^A^	0.38 + 0.01b	0.37 + 0.02b	0.39 + 0.01b	0.38 + 0.01b	0.41 + 0.01a
RS	TC^A^	28.44 + 0.81a	26.14 + 0.68b	26.74 + 0.52b	26.76 + 0.35b	27.03 + 0.64b
	SOM^A^	22.74 + 0.98b	15.6 + 1.35c	22.56 + 0.74b	24.68 + 0.77a	25.55 + 0.85a
	AP	15.28 + 5.04a	13.43 + 1.22a	9.28 + 5.45a	15.35 + 4.93a	13.63 + 2.03a
	pH^A^	7.77 + 0.07ab	7.86 + 0.1a	7.9 + 0.06a	7.9 + 0.1a	7.58 + 0.2b
	Salt^A^	2.15 + 0.24c	2.4 + 0.04bc	2.71 + 0.17b	3.52 + 0.45a	3.38 + 0.3a
	TP^B^	0.66 + 0.02a	0.67 + 0.03a	0.66 + 0.03a	0.65 + 0.03a	0.71 + 0.05a
	TN^B^	0.38 + 0.02a	0.36 + 0.02a	0.38 + 0.01a	0.36 + 0.01a	0.36 + 0.02a

BS, non-rhizosphere soil. RS, rhizosphere soil. Different lowercase letters represent significant differences between treatments. Different capital letters represent significant differences between rhizosphere and non-rhizosphere soil.

### 3.6 Correlation analysis of bacterial community and soil environmental factors

**Figure 5:**
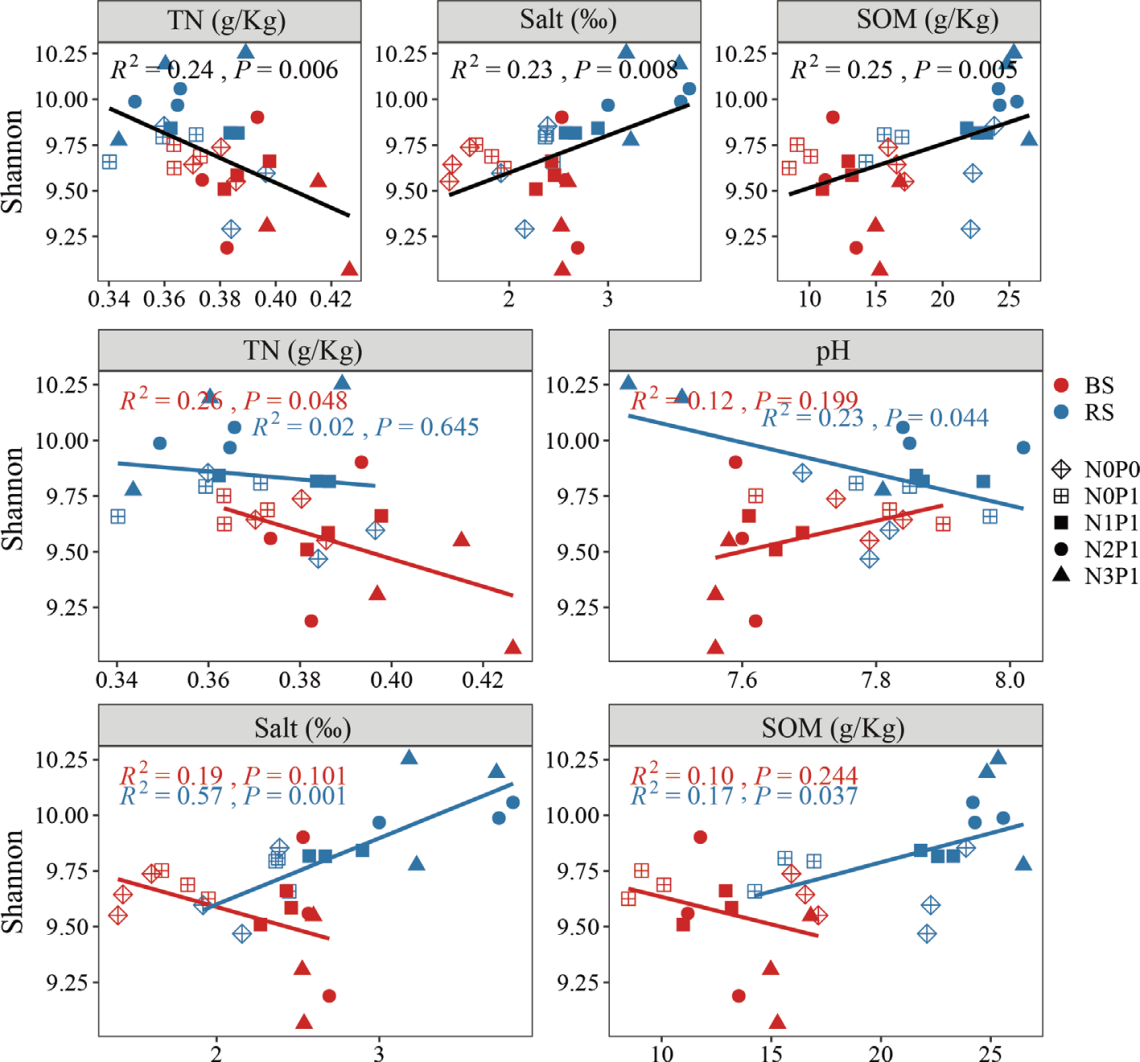
Correlation analysis of Shannon index with soil environmental factors BS, non-rhizosphere soil. RS, rhizosphere soil.

The ABT analysis (**Table S6**) showed that SOM, TN and salt content had the greatest effect on bacterial community diversity. There was a significant positive correlation between the Shannon index of soil bacterial community and soil salt and SOM (**Figure 5**), and significant negative correlation with TN (P < 0.05). In the rhizosphere soil, the Shannon index of the bacterial community increased with increasing soil salt and SOM content, but decreased with increasing pH. The Shannon index of the bacterial community was negatively correlated with the TN content in the non-rhizosphere soil.

**Figure 6:**
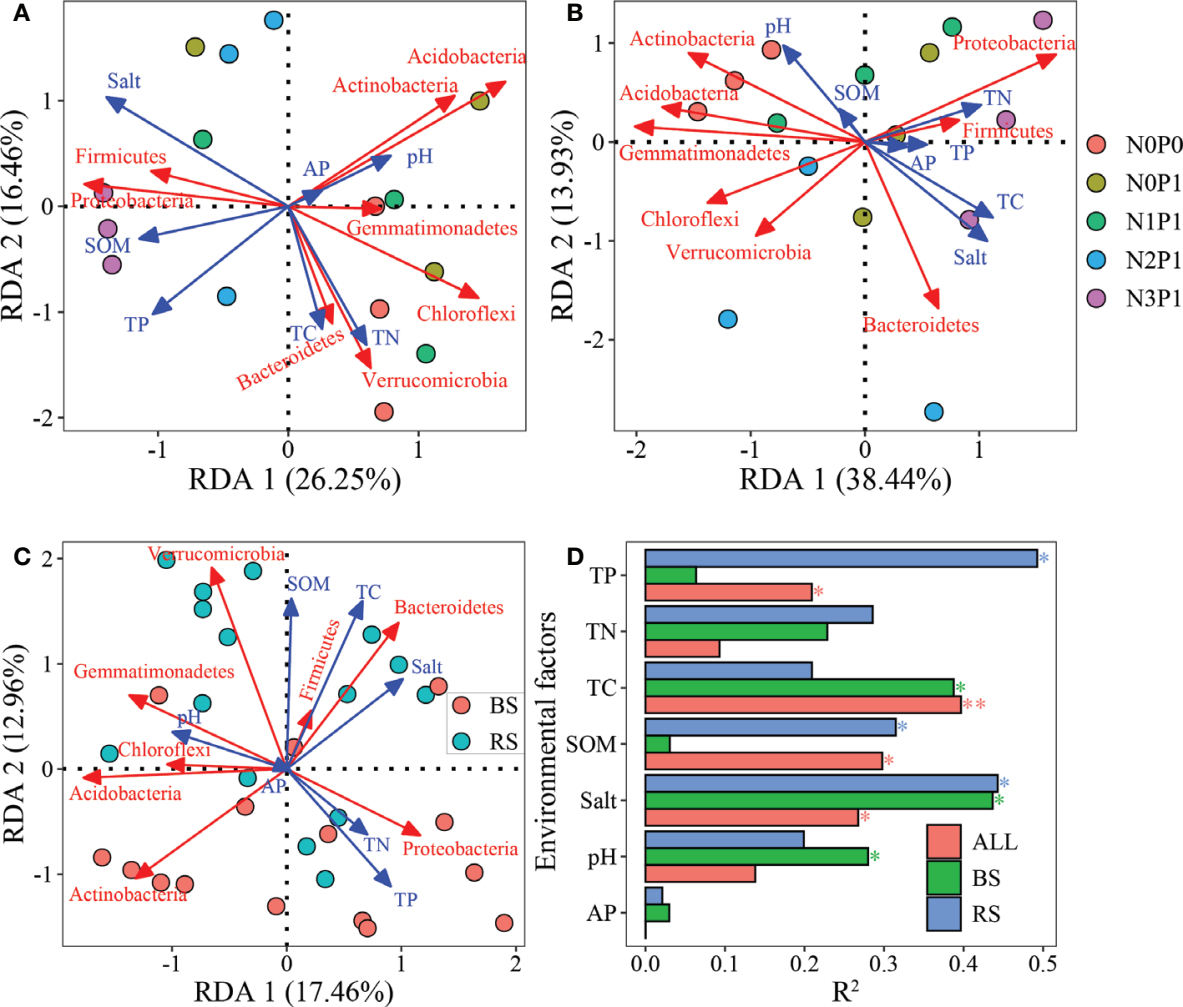
RDA analysis of dominant phyla composition with soil environmental factors. **(A)**, rhizosphere soil. **(B)**, non-rhizosphere soil, **(C)**, both of rhizosphere and non-rhizosphere soil. **(D)**, Contribution of soil environmental factors to variation in bacterial community. BS, non-rhizosphere soil. RS, rhizosphere soil. * and **indicate that the effect of environmental factors is significant at 0.05 and 0.01 level, respectively. RS: rhizosphere soil. BS: non-rhizosphere soil. ALL: rhizosphere and non-rhizosphere soils.

In the rhizosphere soil (**Figure 6A**), the first two axes explained 42.9% of the variation in the bacterial community dominant phyla. The effect of TP, salt and SOM content on the structure of the bacterial community dominant phyla was significant, explaining 49.25%, 44.28% and 31.38% of the variation, respectively (P < 0.05). The bacterial community dominant phyla composition in non-rhizosphere soil (**Figure 6B**) was significantly affected by soil salt, TC and pH (P < 0.05). In both soils, TC, TP, salt and SOM content significantly affected the bacterial community (**Figure 6C**), explaining 39.68%, 20.91%, 26.74% and 29.77% of the variation in the bacterial community dominant phyla composition, respectively (P < 0.05). Soil salt had a significant effect on the bacterial community in all three RDA analyses (**Figure 6D**, P < 0.05). RDA analysis showed a negative correlation between soil salt and both *Actinobacteria* and *Acidobacteria*.

**Figure 7:**
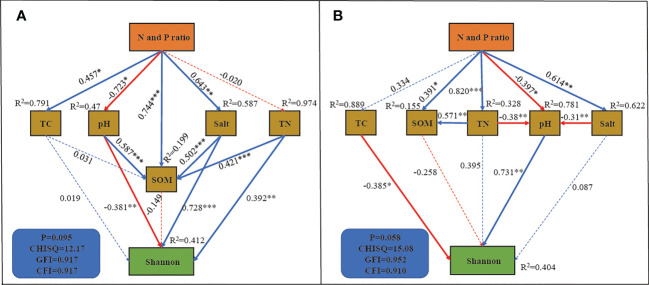
SEM analysis of N and P addition ratio on Shannon index. **(A)**, rhizosphere soil. **(B)**, non-rhizosphere soil. *, **, *** indicate that the path coefficient is significant at 0.05, 0.01 and 0.001 level, respectively.

To further investigate the effect of the N and P addition ratio on the bacterial community, we constructed SEM with good fitness (**Figure 7**). In rhizosphere soil, SEM explained 41.2% of the variation in the Shannon index of the bacterial community (**Figure 7A**). Shannon diversity was directly influenced by pH, Salt and TN content with path coefficients of -0.38, 0.728 and 0.392, respectively. There was a significant positive effect of N and P addition ratio on soil TC, SOM and soil salinity, and a negative effect on pH (P < 0.05). The N and P addition ratio increased bacterial community α-diversity by decreasing pH and increasing soil salinity, with a total effect of 0.275 and 0.468 for the two pathways, respectively. In non-rhizosphere soil, SEM explained 40.4% of the variation in the bacterial community (**Figure 7B**). The Shannon index is directly affected by TN and pH, with path coefficients of -0.385 and 0.731, respectively. The N and P addition ratio indirectly decreased the α-diversity by directly and indirectly decreasing soil pH, with a total effect of -0.518.

## 4 Discussion

### 4.1 Effect of N and P addition ratio on the α-diversity

Plant-bacterial community interactions are regulated by several factors. N and P are among the important regulatory factors (Wang et al., 2018b). Previous studies have found differences in the effects of N addition on the bacterial community without separating rhizosphere and non-rhizosphere soils (Ling et al., 2017; Zhang et al., 2017). In this work, the rhizosphere and non-rhizosphere soil were studied separately and differences were found in the response of α-diversity of the two soils bacterial community to the N-P addition ratio, which is consistent with the results of Wang et al. (2022a) and Ran et al. (2021) et al. On the one hand, plants uptake soil nutrients and buffer the direct effect of fertilization on bacterial community (Wang et al., 2019). On the other hand, plants are influenced by N and P addition and the increase of rhizosphere secretion released indirectly affects the bacterial community (Liu et al., 2016).

In this work, *P.communis* rhizosphere bacterial community α-diversity increased with increasing N and P addition ratios, while non-rhizosphere bacterial community α-diversity decreased. SEM analysis showed that N and P addition ratio indirectly decreased α-diversity by lowering pH in non-rhizosphere soil, which is consistent with previous studies (Chen et al., 2021; Niu et al., 2021). Although the N and P addition ratio increased the non-rhizosphere soil TC content, it did not have a significant increase on bacterial community α-diversity. Ning et al. (2021) found that N addition increased soil TC but reduced the effectiveness of soil microbial available C, leading to increased microbial C limitation and inhibition of microbial activity. Unlike in non-rhizosphere soil, N and P addition decreased soil pH and increased soil salt to increase rhizosphere soil bacterial α-diversity. Yao et al. (2021) found that soil salt and pH dominated the soil bacterial community diversity in saline agricultural fields, which is consistent with our study. pH was significantly lower in the N3P1 treatment, but was higher and less variable compared to non-interstitial soil. Brigham et al. (2022) found that N addition only resulted in lower soil pH when the plant was removed, which is consistent with our study.

Salt is an important factor in changing the bacterial community (Kamble et al., 2014; Wang et al., 2018a). Increased salt content affects plant growth and bacterial community diversity and structure(Cheng et al., 2021), negatively affecting ecosystem stability, while lower soil salinity favors bacterial community richness and diversity in coastal wetland ecosystems(Li et al., 2021). The increase in rhizosphere soil bacterial community α-diversity was mainly due to the increase in soil salt, which was inconsistent with most previous studies. *P.communis* are typically salt-rejecting plants (Zhao et al., 2022a), and the root can filter salt ions to reduce the damage. The N and P ratio increased water uptake, leading to an increase in rhizosphere soil salt. Contrary to conventional wisdom, increasing salt does not simply decrease bacterial diversity, but increases microbial abundance and diversity at moderate salt levels (Ji et al., 2019). This indicates that despite the increase in *P.communis* rhizosphere soil salt, it did not reach the threshold for suppression of bacterial community α-diversity. The TN content of non-rhizosphere soil increased with the increase of the N-P input ratio, and there was a significant negative correlation with bacterial community diversity. However, in rhizosphere soil, TN content did change insignificantly with increasing N and P ratios. Zhao et al. (2022b) found that *P.communis* absorb as much soil N as possible and stores it in their body to provide for their growth. SEM showed a significant positive effect of TN content on α-diversity, which may be caused by the N limitation of the rhizosphere bacterial community due to the uptake of soil N with *P.communis*.

### 4.2 Response of bacterial community structure to N and P addition ratio

Soil and plant properties and plant-microbe interactions affect bacterial community composition(Weand et al., 2010). PCoA analysis showed that the N and P addition ratio had insignificant effects on the bacterial community structure of the rhizosphere soil and significant effect on that of non-rhizosphere soil. The difference in variance between treatments increased with increasing N and P addition ratio, which is similar to the previous study(Ling et al., 2017). While in the rhizosphere soil, only the N3P1 and N0P1 treatments were different from the N0P0. Plant growth in the Yellow River Delta is mainly N-limited (Zhang et al., 2015), and P addition exacerbates the N-limitation of *P.communis* growth. Response of plants to N limitation altered bacterial community structure. In addition, the P addition decreases the N/P of the root secretion, thus causing major variations in the bacterial community structure(Ma et al., 2023). PERMANOVA analysis revealed differences in the effects of both N and P on the bacterial community structure of the two soils, which may be due to *P.communis* buffering soil nutrient changes (Wang et al., 2019).

### 4.3 Response of bacterial community phylum composition to N and P addition ratio

In the present study, the highest relative abundance of *Proteobacteria* was found in both soils, which is consistent with previous reports (Li et al., 2018; Shao et al., 2019), suggesting that *Proteobacteria* is more widely adapted. The relative abundance of *Acidobacteria* and *Actinobacteria* was highest in the N0P0 treatment, while the relative abundance decreased with the increase of the N-P addition ratio. The N and P addition ratio increased the relative abundance of the non-rhizosphere soil *Proteobacteria* and *Bacteroidetes*, which was similar to the previous studies (Ma et al., 2022; Ma et al., 2023). *Proteobacteria* and *Bacteroidetes* are eutrophic taxa (Wang et al., 2022b), and soil nutrients increase the relative abundance of both. The *Acidobacteria* belongs to oligotrophic taxa, and increased nutrients hurt their abundance (Sha et al., 2021). *Actinobacteria* belong to eutrophic taxa, however, nutrient addition had a significant negative effect on their abundance (Li et al., 2019b). The RDA analysis showed negative correlation between *Actinobacteria* and salt. *Actinobacteria* are susceptible to salt limitation (Zhao et al., 2020). In the rhizosphere soil, the relative abundance of the *Chloroflexi* decreased with increasing N and P ratios. A study (Hu et al., 2022) showed that N addition reduced the relative abundance of *Chloroflexi*. The *Chloroflexi* uses CO_2_ as a C source for photosynthesis (Klatt et al., 2013). Elevated N and P ratio increases SOM content, which may be at a disadvantage in competition with other bacteria (Yang et al., 2022b). The relative abundance of *Bacteroidetes* of rhizosphere soil was higher than that of *Actinobacteria* because *Bacteroidetes* have stronger salt tolerance and are more adaptable to the high salt environment of *P.communis* rhizosphere soil (Zhao et al., 2020).

### 4.4 Effect of plants on bacterial community

Symbiotic networks are increasingly used to explore potential microbial interactions (Wang et al., 2022a). In this study, it was found that the number of nodes, the number of edges and the complexity of the symbiotic network of rhizosphere soil were lower than those of non-rhizosphere soil, which is consistent with the previous study (Ling et al., 2022). Modularity of the rhizosphere soil bacterial network was higher than that of the non-rhizosphere soil, but the bacterial symbiotic network in the rhizosphere soil was less stable (Ling et al., 2022). Wang et al. (2022a) found that network complexity was significantly influenced by plant root secretions. Compared to non-rhizosphere soil, the rhizosphere environment is influenced by plant secretions and has a stronger ability to select for the bacterial community. In addition, the increase of N and P addition ratio may strengthen the ability of the rhizosphere environment to select bacterial community, which is detrimental to the robustness of the network. Annotation of key nodes in the symbiotic network revealed that *Longimicrobiales* order, *Pseudomonas* order, *Cyclobacteriaceae* family and *MND1* genera were significantly higher in the rhizosphere than in non-rhizosphere soil. Qian et al. (2022) found that the abundance of *Longimicrobiales* order was higher in rhizosphere soil than in non-rhizosphere soil, due to more dodecanoic acid in rhizosphere soil. Yang et al. (2022a) found that the *Longimicrobiales* order is mainly associated with root apoplast through the modular function of the co-occurrence network, which further corroborates its close connection with plant roots. *Pseudomonas* has been shown to suppress plant pathogens and promote plant growth (Tao et al., 2020). In addition, Pseudomonas has a strong N fixation capacity (Li et al., 2022b) and can provide N for *P.communis*, which may also be an important reason why *P.communis* can adapt to N-deprived habitat. Both *the Cyclobacteriaceae* family and *MND1* genera were also found to promote plant growth (Li et al., 2020; Cheng et al., 2022), which is consistent with our study.

## 5 Conclusion

Increased N and P input ratio reduced the non-rhizosphere bacterial communityα-diversity, which was caused by the decrease of pH. The rhizosphere soil bacterial community α-diversity increased with increasing N and P ratios. This was due to the absorption of N from the soil by the *P.communis*, which reduced the direct effect of N and P addition on the bacterial community. Variation in rhizosphere soil bacterial community α-diversity is due to plant response to N and P addition ratio. The effect of imbalance N and P addition ratio on the community structure of rhizosphere bacteria was less than that of non-rhizosphere soil. N and P additions increased the relative abundance of *Bacteroidetes* and *Proteobacteria* in non-rhizosphere soil and decreased the relative abundance of *Acidobacteria* and *Actinobacteria*. Salt is a key influencing factor in the composition of both soil bacterial communities. These results can deepen our understanding of the relationship between plant and bacterial community and provide theoretical guides for the restoration of degraded soil in coastal wetlands under the background of N deposition.

## Data availability statement

The datasets presented in this study can be found in online repositories. The names of the repository/repositories and accession number(s) can be found below: https://www.ncbi.nlm.nih.gov/, PRJNA935937.

## Author contributions

ZZ completed data analysis and wrote the manuscript. JS conceived and designed the study, and fund support. TL and PS were in charge of the modification of the manuscript. JM and KD conducted sample collection. All authors contributed to the article and approved the submitted version.
