# Supplementary material for: Plants changed the response of bacterial community to the nitrogen and phosphorus addition ratio
Source: Front Plant Sci. 2023 Mar 27;14:1168111. doi: 10.3389/fpls.2023.1168111 (PMC10083283; doi:10.3389/fpls.2023.1168111)
Supplement: Supplementary file 1 [file DataSheet_1.docx]

Plants changed the response of bacterial community to the nitrogen and phosphorus addition ratio

Zehao Zhang^1,2^, Jingkuan Sun^1*^, Tian Li^1^, Pengshuai Shao^1^, Jinzhao Ma^1^, Kaikai Dong^1^

*^1^Shandong Key Laboratory of Eco-Environmental Science for Yellow River Delta, Binzhou University, Binzhou, China*

*^2^College of Forestry, Shandong Agricultural University, Taian, China*

*** Correspondence:**Jingkuan Sun
sunjingkuan@126.com

**TABLE S1 | ANOVA analysis of α-diversity of bacterial community between rhizosphere and non-rhizosphere soil in same treatment.**

| Treatment | Chao1 | |  | Shannon | |
| --- | --- | --- | --- | --- | --- |
|  | *F* | *P* |  | *F* | *P* |
| N0P0 | 6.431 | 0.064 |  | 0.138 | 0.729 |
| N0P1 | 1.519 | 0.285 |  | 1.175 | 0.339 |
| N1P1 | 4.533 | 0.100 |  | 28.922 | 0.006 |
| N2P1 | 9.222 | 0.039 |  | 4.779 | 0.094 |
| N3P1 | 15.186 | 0.018 |  | 14.017 | 0.020 |

**TABLE S2 | Multi-factor ANOVA analysis of α-diversity of bacterial community.**

| Factors | Chao1 | |  | Shannon | |
| --- | --- | --- | --- | --- | --- |
|  | *F* | *P* |  | *F* | *P* |
| N | 2.079 | 0.135 |  | 0.463 | 0.711 |
| P | 0.106 | 0.748 |  | 0.979 | 0.334 |
| Soil position | 13.353 | 0.002 |  | 17.807 | 0.001 |
| N×Soil position | 13.239 | 0.001 |  | 5.888 | 0.005 |
| P×Soil position | 0.672 | 0.422 |  | 0.344 | 0.564 |

**TABLE S3 | PERMANOVA analysis of bacterial community structure in rhizosphere and non-rhizosphere soil, respectively.**

| Soil position | Factors | *R* | *P* |
| --- | --- | --- | --- |
| RS | N | 0.238 | 0.140 |
|  | P | 0.087 | 0.135 |
|  | Residual | 0.674 |  |
| BS | N | 0.492 | 0.001 |
|  | P | 0.135 | 0.001 |
|  | Residual | 0.373 |  |

**Notes: BS, non-rhizosphere soil. RS, rhizosphere soil.**

**TABLE S4 | PERMANOVA analysis of bacterial community structure in all soils.**

| Factors | *R* | *P* |
| --- | --- | --- |
| N | 0.163 | 0.001 |
| P | 0.040 | 0.037 |
| Soil position | 0.151 | 0.001 |
| N×Soil position | 0.153 | 0.001 |
| P×Soil position | 0.056 | 0.004 |
| Residual | 0.438 |  |

**TABLE S5 | Sub-network topology of rhizosphere and non-rhizosphere bacterial community.**

|  | Nodes | Edges | Edge density | Degree centralization | Average clustering coefficient | Complexity | Modularity |
| --- | --- | --- | --- | --- | --- | --- | --- |
| RS | 117 | 684 | 0.100795756 | 0.24403183 | 0.587035085 | 5.846153846 | 0.414653 |
| BS | 125 | 825 | 0.106451613 | 0.256451613 | 0.630491907 | 6.6 | 0.373789 |

**Notes: BS, non-rhizosphere soil. RS, rhizosphere soil.**

**TABLE S6 | Aggregated boosted tree (ABT) of rhizosphere and non-rhizosphere bacterial community.**

| Soil environmental factors | Relative influence(%) | Rank |
| --- | --- | --- |
| SOM | 21.57038 | 1 |
| TN | 20.56138 | 2 |
| Salt | 18.24689 | 3 |
| TC | 16.94873 | 4 |
| pH | 10.55953 | 5 |
| TP | 7.771933 | 6 |
| AP | 4.341159 | 7 |

**FIGURE S1 | Relative abundance of key OTUs in symbiotic networks**


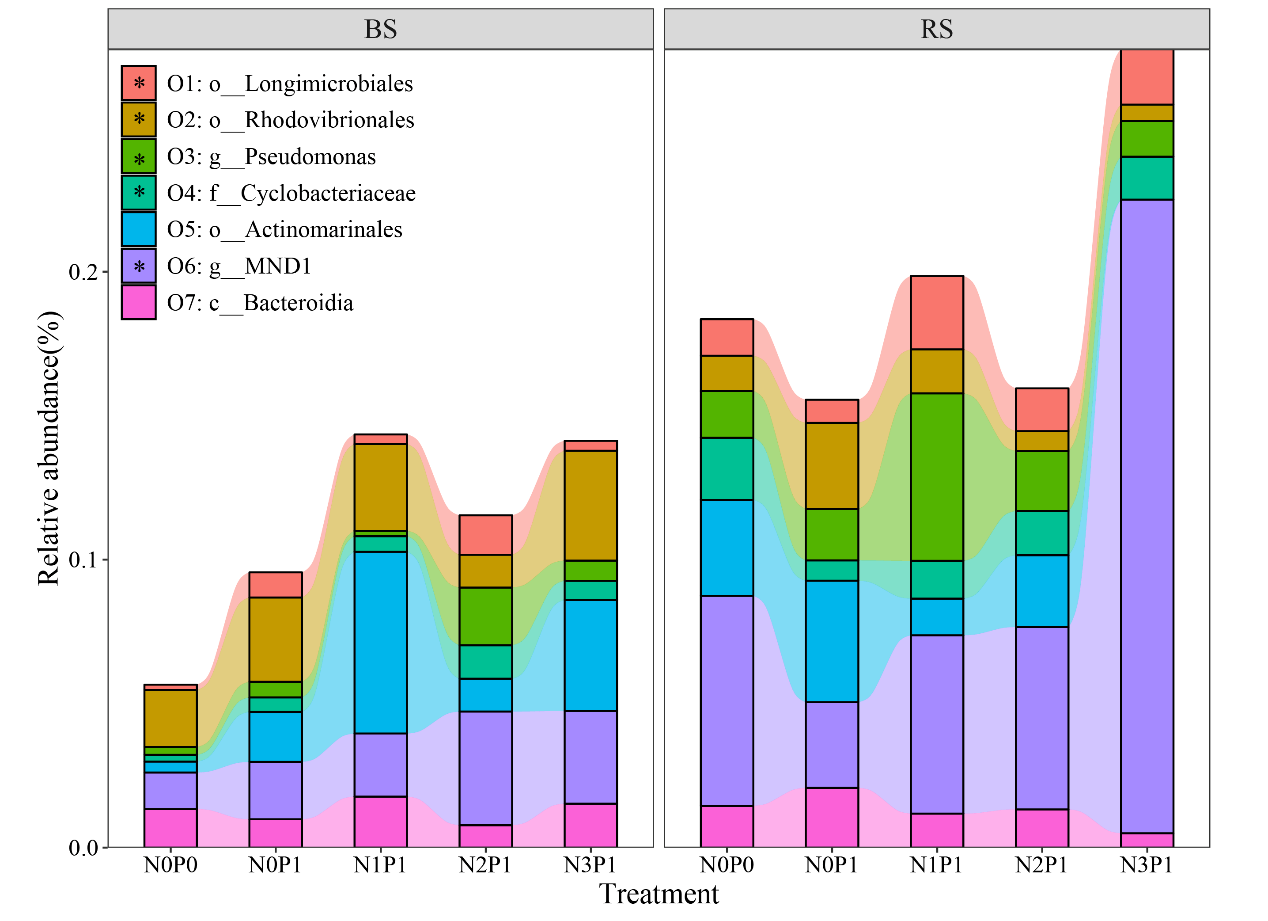


**Notes: BS, non-rhizosphere soil. RS, rhizosphere soil. * represents significant difference between rhizosphere and non-rhizosphere soil.**
